# Can public health services promote the settlement intention of migrant workers: empirical analysis from China

**DOI:** 10.3389/fpubh.2024.1472223

**Published:** 2024-11-20

**Authors:** Tong Lyu

**Affiliations:** School of Humanities and Arts, China University of Mining and Technology, Xuzhou, China

**Keywords:** public health services, health record, public health education, migrant worker, settlement intention

## Abstract

**Introduction:**

Enhancing migrant workers’ settlement intention in cities requires ensuring they have equal public health rights as urban residents. Full access to public health services can strengthen their sense of belonging and improve the well-being of this vulnerable group. Evaluating the welfare impact of public health services from the perspective of city identification offers valuable insights and informs policies aimed at improving the quality of public health service provision.

**Methods:**

This study utilizes data from the 2017 China CMDS survey. We employed various analytical methods, including the Probit model, IV-Probit model, Propensity Score Matching, and KHB decomposition, to empirically examine the impact of public health services on the settlement intention of migrant workers. Additionally, we explored the underlying mechanisms and heterogeneity of this impact.

**Results:**

Public health services such as health records management and public health education significantly increase the settlement intention of migrant workers. The positive effect of public health services on the settlement intention is more pronounced among migrant workers who have moved across provinces and those who are married. Public health services indirectly enhance the settlement intention by improving urban satisfaction and sense of belonging, with the latter having a more substantial indirect effect.

**Discussion:**

The current provision of basic public health services in China for migrant workers still needs improvement. This highlights the necessity of enhancing health record management, increasing health education and training, and tailoring services to better meet the needs of migrant workers. By improving the supply of public health services, we can effectively raise migrant workers’ urban satisfaction and sense of belonging, thereby indirectly increasing their willingness to settle in cities. The findings of this study contribute to further optimizing the implementation of public health service policies and provide meaningful guidance for improving the urban integration of migrant workers.

## 1 Introduction

In China, the rapid urbanization and industrialization since the reform and opening-up have led to a widening gap between urban and rural areas, prompting millions of migrant workers to move between these regions. In a broad context, migrant workers refers to individuals who leave their hometowns or places of origin to seek employment or job opportunities in other regions or countries. In China, the term typically refers to rural laborers who migrate to cities for manual or low-skilled work (1). According to the results of the Seventh National Population Census, by 2021, the number of migrant workers in China reached 375.82 million, accounting for approximately one-quarter of the total population (2). Migrant workers have become a crucial component of China’s labor market. According to data from the National Bureau of Statistics, there were 176.58 million migrant workers in China in 2023, an increase of 4.68 million or 2.7% compared to the previous year (3). Among them, 67.51 million migrant workers, accounting for 38.2% of the total internal migrant population moved across provinces. Meanwhile, 109.07 million workers, or 61.8%, migrated within their own provinces. However, due to China’s rigid household registration (Hukou) system, most social welfare policies are based on this system, creating constraints under the dual urban–rural structure (4). As a crucial residency document for Chinese citizens, the hukou is an official record issued by the government that verifies an individual’s legal residence in a specific area (5). As a result, migrant workers cannot fully enjoy the same social benefits as local residents. This suggests that public health services including programs like the establishment of health records and health education, were traditionally offered as a form of social welfare primarily to urban residents. Migrant workers often face significant barriers when accessing public health services (6). These barriers to accessing benefits have resulted in a “migratory bird” pattern of migration for many migrant workers, rather than permanent relocation. Consequently, the overall willingness of migrant workers to integrate into urban life remains low (7). Ensuring that public health services benefit the vast migrant worker population is a critical yet weak link in the construction of China’s public health service system.

Improving the quality and degree of urbanization to promote healthy and stable urban development is a significant challenge faced by governments worldwide (8). Since the implementation of the reform and opening-up policy, a milestone in China’s urbanization process, the country has successfully established a market economy. This has led to the migration of hundreds of millions of people from rural areas to cities. According to the National Bureau of Statistics, China’s urbanization rate increased from 17.92% in 1978, at the start of the policy, to 66.16% in 2023, with an average annual growth of 1.07%. By the end of 2023, the number of cities nationwide had reached 694, with a total urban population of 673.13 million. Among these, 29 cities had populations exceeding 5 million, and 11 cities had populations over 10 million (9). The core of urbanization lies in the mindset and identity transition of internal migrants. As the primary agents of China’s current urbanization process, migrant workers’ willingness to settle in urban areas is a key factor in determining the success of urbanization. Multi-faceted urbanization policies, aimed at improving the welfare of migrant workers, play a crucial role in shaping their settlement intentions. The settlement intention of migrant workers refers to their desire to become long-term residents of the cities where they work. To enhance migrant workers’ settlement intentions, the Chinese government has been continuously advancing reforms in basic public health services. Since the implementation of the “New Medical Reform” policy in 2009, a series of documents, such as the “Opinions of the Central Committee of the Communist Party of China and the State Council on Deepening the Reform of the Medical and Health System” and the “12th Five-Year Plan for the Development of Health Services,” have been issued. These policies emphasize the importance of public health services for migrant workers, aiming to improve their sense of gain and settlement intentions by providing equitable public health services. Despite the expansion of public health services available to migrant workers in their destination cities, their willingness to settle permanently has been steadily declining. This declining settlement intentions has become a new challenge in the urban integration of migrant workers. While the national government places significant emphasis on the top-level design of public health services and has rapidly advanced their implementation, research on the role of public health services in the urbanization process of migrant workers has lagged behind.

The marginal contributions of this paper are reflected in three aspects: First, this paper utilizes data from the China Migrants Dynamic Survey (CMDS), organized by the National Health Commission of China, to systematically evaluate the impact of basic public health services on the willingness of migrant workers to remain in cities. It focuses on two aspects: public health education and health records management, and analyzes the underlying mechanisms at play. Second, this study provides new empirical evidence on how specific public health service measures can improve the living conditions of migrant workers and advance their urban integration process. Third, the findings of this study contribute to understanding the development process of equalizing public health services and offer meaningful theoretical guidance for promoting the equalization of public health services for the migrant population.

## 2 Literature review and research hypothesis

### 2.1 Literature review

Entering the 21st century, the number of migrant workers leaving their hometowns to work in cities has steadily increased, driven by China’s deepening industrialization process. As the most important human resource in the current labor market, the settlement intentions of migrant workers is crucial for China’s urbanization and urban development. Understanding the driving factors behind their settlement intentions is essential for formulating economic development policies. Despite substantial research on this topic, there is still debate regarding the determinants of migrant workers’ settlement intentions. Theoretical studies often rely on economic or sociological frameworks to understand labor migration patterns and explore the historical development and future trends of migrant workers’ urban integration. Specifically, previous research has used various theoretical frameworks such as immigration economics theory (10), labor market segmentation theory (11), neoclassical economics theory (12), spatial difference theory (13, 14), and income disparity theory (15). These studies have examined the institutional characteristics of the environments in which migrant workers live, individual characteristics, and migration characteristics as explanatory variables in a comprehensive analysis. On the empirical research level, past studies have explored various dimensions such as urban environmental quality (16), economic development policies (17), degree of urban integration (18), communication technology (19), living conditions of the migrant population (20, 21), economic status and public service (22). These factors have all been shown to be associated with the settlement intentions of migrant workers to varying degrees. Public services, as the core and essence of government functions and an important reflection of government responsibility, have a significant impact on migrant workers’ willingness to settle in cities. This influence has been a focal point of numerous studies. Previous research has examined the effect of public services on migrant workers’ settlement intentions from both micro and macro perspectives. The study covers various service areas, including regional talent allocation, technology business incubation, cross-regional collaboration, higher education investment, and public health services (23). As a vital component of public services, public health services are a key pathway to achieving universal health coverage (24). When research is focused specifically on public health services, most existing studies concentrate on several key areas: the progress of public health service implementation, the various factors influencing the equalization of public health services, and the impacts these services have (25, 26).

Existing literature on the factors influencing migrant workers’ willingness to settle in cities often focuses on public services such as labor protection, basic education, social insurance, and infrastructure development. These studies recognize the importance of these public services in enhancing the settlement intentions of the migrant population and promoting their urban integration. However, the impact of public health services is frequently overlooked or only included as a control variable in the analysis. Systematic research specifically targeting public health services is rare. Existing studies that treat it as an explanatory variable largely focus on the fairness of policy promotion and implementation (27). Some empirical studies have examined the impact of public health services on various aspects such as labor supply, the utilization level of medical services, health status, and capabilities (28, 29, 30, 31). From a core objective perspective, the fundamental value of public health services lies in ensuring that every resident has access to essential services for protecting and maintaining their health (32). Previous studies have explored the priorities of various countries regarding the value objectives of public health services. For instance, some countries focus more on balancing the quantitative allocation of healthcare within the public health service process (33). Long-term practice in public health services has led China’s government health departments to recognize that providing high-quality and equitable public health services to all residents, including migrants, is of utmost importance (34). Migrant workers, as key participants and contributors to China’s economic and social development, have nonetheless been marginalized in terms of access to public health services in previous research (35). For a long time, migrant workers in China have had relatively weak public health awareness and are more susceptible to diseases, making them a “vulnerable” group in health risk management (36). Therefore, the quality of public health services available to this group deserves greater attention.

### 2.2 Theoretical analysis and research hypothesis

Migration theory posits that institutional adjustments and the implementation of public policies are significant factors influencing population migration (37). Among these factors, the construction of institutions and implementation of policies related to public health resources have a significant impact on the institutional trust and settlement intentions of the migrant population in the applicable areas. According to institutional theory, public political trust stems from their confidence in the political system and their assessment of the system’s performance (38). Within the existing institutional framework, government governance performance encompasses various aspects, including economic, social, and public resource provision. One crucial element is the accessibility of public health resources to individuals, which reflects the extent to which individuals can benefit from these resources (39). In fact, striving to achieve the equalization of public health resources has been a policy trend of the Chinese government in recent years, a trend that has become particularly prominent since 2009 (40). Equalization refers to the principle that the migrant population should enjoy public health services provided by the government on the same basis as the local registered population, without any differences (41). The ultimate goal of public health resource provision is to balance efficiency and equity, ensuring that all members of society have as fair access to public resources as possible (42). This accessibility is a rational reflection of governance performance from the subjective perspective of citizens. The essence of urban integration lies in granting migrant workers equal social rights, with access to public health services being one of the most fundamental of these rights. A key prerequisite for achieving this outcome is that migrant workers, as the primary group within the floating population, possess a desire for urban integration. This desire is rooted in their settlement intentions permanently. Migrant workers’ settlement intentions are closely tied to the household registration hukou system. China’s relatively rigid hukou system leads to significant variation in their intention to change residency status, as different regions impose different registration barriers (43). A reasonable policy approach, therefore, would be to optimize the provision of public health services to mitigate the impact of the household registration system. This would better protect the health rights of migrant workers and, in turn, increase their settlement intentions.

What is the mechanism through which public health service provision influences migrant workers’ willingness to settle in cities? As a public service provided by the government, public health services were originally restricted by the household registration system. In the past, these services primarily focused on prioritizing the local registered population (44). The goal of optimizing public health service provision is to overcome the limitations imposed by the household registration system. This approach extends social rights to migrant workers, effectively allowing a partial transfer of social rights typically reserved for the local registered population (45). This transfer of social rights reflects the city’s recognition of migrant workers as local social citizens. It helps reduce the psychological distance between migrant workers and the city, strengthens their sense of belonging and identification with urban life, and increases their willingness to remain in the city. A possible mechanism is that public health services foster a stronger sense of urban satisfaction among migrant workers, thereby increasing their settlement intentions. This identification typically encompasses various aspects, such as urban satisfaction and urban belonging (46). This perception of urban satisfaction reflects an individual’s overall feelings about various aspects of their city’s environment, including infrastructure, public services, and cultural values (47). According to the Theory of Planned Behavior, individuals make rational decisions based on their attitudes, subjective norms, and perceived behavioral control (48). According to the Theory of Planned Behavior, migrant workers’ settlement intentions depends on their attitudes (city preference), subjective norms (identity recognition), and perceived behavioral control (migration motivation). Migration motivation directly influences their migration behavior, while city preference and identity recognition impact their behavior through their sense of identification with the city they move to (49). Settlement intention is constrained by this sense of identification. Migrant workers who have higher satisfaction with the city and a stronger sense of belonging are more likely to settle in urban areas. Based on this analysis, this paper proposes the following research hypothesis:

H1: Public health services significantly increase the willingness of migrant workers to settle in cities.

H2: Public health services enhance the settlement intention of migrant workers by improving their urban satisfaction and urban belonging.

## 3 Materials and methods

### 3.1 Study design and data sources

This study utilizes data from the China Migrants Dynamic Survey (CMDS), which is an annual large-scale national survey of the migrant population organized by the National Health Commission since 2009. The survey includes migrant individuals aged 15 and above who have lived in their destination for more than 1 month and do not have local household registration, covering all 31 provinces, municipalities, and autonomous regions in China. The survey encompasses various aspects of the migrant population and their family members, including migration patterns and trends, health status, health record establishment, employment, income and housing, and marital status. For this study, we used data from the 2017 CMDS. The survey used the 2017 annual population mobility report data from all 31 provinces (autonomous regions, municipalities) and the Xinjiang Production and Construction Corps as the basic sampling frame. A stratified, multi-stage, probability proportional to size (PPS) sampling method was employed. The sample distribution covers 1,459 county-level areas, 3,776 townships, and 8,993 neighborhood committees across various provinces, cities, and counties nationwide, with a total of 169,989 survey responses. Given that the focus of this study is on migrant workers, only the agricultural household registration population that has migrated for work is retained. After excluding samples with missing key information, the final full sample includes 79,715 observations of migrant workers.

### 3.2 Variable measurement

#### 3.2.1 Dependent variables: settlement intention

The dependent variable in this study is the willingness of migrant workers to settle in cities. We measure this using the 2017 CMDS questionnaire item from the “Migration and Settlement Intentions” section, which asks, “Do you plan to continue staying here for the next period?” The questionnaire provides six response options: “1–2 years,” “3–5 years,” “6–10 years,” “more than 10 years,” “settle permanently,” and “undecided.” Following previous research (50), we define the assignment rule as follows: if respondents are willing to stay in their current location for more than 5 years (including the options “6–10 years,” “more than 10 years,” and “settle permanently”), the settlement intention variable is assigned a value of 1; otherwise, it is assigned a value of 0. According to the sample data used in this study, 48.52% of respondents indicated a willingness to stay in their current location for more than 5 years.

#### 3.2.2 Independent variables: public health services

Public health services are the core explanatory variable in this study. According to the “Pilot Work Plan for Equalization of Public Health Services for Migrant Populations,” issued in 2013 by the former National Health and Family Planning Commission, basic public health services for migrant populations include multiple components: public health education, health records management, vaccination for migrant children, and maternal and child health management for migrant women and children. Referencing previous research (51) and considering the actual public health services provided by grassroots medical institutions, this study uses the establishment of health records and receipt of health education as proxy variables for public health services. In the questionnaire, the question regarding the establishment of health records is “Has a resident health record been created for you in this location?” Responses are coded as 1 for “Yes, established” and 0 for all other answers. The question regarding receipt of health education is “In the past year, have you received any of the following public health education topics in the destination location: nutritional health knowledge, occupational disease prevention, smoking control, mental disorder prevention, infectious disease prevention, etc.?” If respondents received at least one type of health education, they are considered to have received public health education, coded as 1; otherwise, they are coded as 0. Among the respondents, 73.85% received at least one type of health education, but only 29.53% had established health records.

#### 3.2.3 Controlled variables

This study, based on the 2017 CMDS survey data and previous research (52), controls for several variables that may influence migrant workers’ settlement intention. These variables include individual characteristics, family characteristics, and migration characteristics. The individual characteristic variables encompass age, gender, and education level. Family characteristic variables include marital status, family size, and average monthly household income. Migration characteristic variables cover the scope of migration and the duration of migration. Descriptions of these variables are presented in Table 1.

**Table 1:** Data definition and descriptive statistics.

Variable	Definition	Mean	SE	Min	Max
Settlement intention	Whether they are willing to live in the local area for 5 years and above: Yes = 1; No = 0	0.485	0.5	0	1
Health records	Whether or not a population health record has been established: Yes = 1; No = 0	0.295	0.456	0	1
Health education	Whether or not receive at least one type of health education: Yes = 1; No = 0	0.738	0.439	0	1
Age	Age in years	37.811	9.1	17	60
Gender	Male = 1; Female = 0	0.572	0.495	0	1
Marital status	Married = 1; Unmarried/Divorced/widowed = 0	0.835	0.371	0	1
Education level	Illiteracy = 1; Elementary school = 2; Middle school = 3; High school/vocational school = 4; 3-year college = 5; 4-year college = 6; Graduate = 7	3.331	1.029	1	7
The flow distance	Cross-provincial mobility = 1; Intra-provincial mobility = 0	0.505	0.5	0	1
The flow time	Time in years	8.084	6.029	2	47
Monthly household income	Income in months	7,255.776	5,421.658	50	200,000
Family size	Size in members	3.254	1.166	1	10

### 3.3 Statistical methods

To assess the impact of basic public health services on the urban settlement intentions of internal migrants, we use Stata 18.0 for the analysis. Given that the dependent variable, settlement intention, is a binary variable, a Probit model is employed for regression analysis. The specific model setup is as follows:

$$\begin{array}{l} \mathrm{Probit}\left(\mathrm{Settlemen}t_{i}=1\right)=\\ F\left(\alpha_{1}+\beta_{1}FILE_{i}+\beta_{2}EDU_{i}+\gamma_{1}\mathrm{CONTRO}L_{i}+\varepsilon \right) \end{array}$$

In this model, ${\mathrm{Settlement}}_{i}$ represents settlement intention, $FILE_{i}$ represents the establishment of health records, $EDU_{i}$ represents the receipt of health education, $\mathrm{CONTRO}L_{i}$ serves as a control variable, ε is the random error term. $\beta_{1}$ is the coefficient representing the impact of establishing health records on settlement intention, $\beta_{2}$ represents the coefficient for the impact of receiving health education on settlement intention, $\gamma_{1}$ is the coefficient for the control variables. This study’s estimation model may face endogeneity issues. The impact of public health services on migrant workers’ urban settlement intentions may involve a self-selection problem. The level of public health services that migrant workers receive depends not only on the public service provision in the destination city but also on the differentiated demand for public health services among migrant workers. Some migrant workers with a strong intention to settle in the city may be more proactive in obtaining public health services. Conversely, individuals who prioritize public service experiences may be more inclined to settle in areas with higher levels of public health service provision. This creates a bidirectional causality problem. Additionally, the estimation model may suffer from endogeneity due to omitted variables. To address these potential endogeneity issues, this study also employs the IV-Probit model and Propensity Score Matching (PSM) model. The IV-Probit model uses effective instrumental variables to handle problems caused by omitted variables, bidirectional causality, and measurement errors. This approach helps mitigate the endogeneity concerns and provides more robust results. PSM is an effective method for reducing confounding effects and addressing potential endogeneity issues in the model (53). In this study, we employ K-nearest neighbor matching, radius matching, and kernel matching methods to jointly examine health effects.

## 4 Empirical results

### 4.1 Baseline regression

The estimation results of the impact of public health services on the settlement intentions of migrant workers, obtained from the probit model regression, are presented in Table 2. Models 1 and 3 in Table 2 show the basic regression results of establishing health records and receiving health education on the settlement intentions of migrant workers, respectively, without including control variables. Models 2 and 4 incorporate individual characteristics, family characteristics, and mobility characteristics as control variables, providing adjusted regression results for the impact of health records and health education on settlement intentions.

**Table 2:** Regression results of factors influencing migrant workers’ settlement intention.

Variables	(1)	(2)	(3)	(4)
Health records	0.155*** (0.010)	0.121*** (0.010)		
Health education			0.103*** (0.010)	0.067*** (0.010)
Age		−0.001 (0.001)		0.001 (0.001)
Gender		−0.001 (0.009)		−0.003 (0.009)
Marital status		0.272*** (0.016)		0.272*** (0.015)
Education level		0.171*** (0.015)		0.171*** (0.005)
The flow distance		−0.377*** (0.009)		−0.380*** (0.009)
The flow time		0.043*** (0.0001)		0.043*** (0.001)
Monthly household income (ln)		0.210*** (0.001)		0.209*** (0.001)
Family size		0.030*** (0.004)		0.030*** (0.005)
Cons	−0.083*** (0.005)	−2.983*** (0.079)	−0.114*** (0.009)	−2.999*** (0.079)
Observations	79,713	79,713	79,713	79,713
Pseudo *R*^2^	0.023	0.068	0.010	0.067

Robust standard errors in parentheses. **p < 0.05, ***p < 0.01.

In Model 1, the regression coefficient for establishing health records is 0.155 (*p* < 0.01). When control variables are added in Model 2, the regression coefficient is 0.121 (*p* < 0.01). For Model 3, the regression coefficient for receiving health education is 0.103 (*p* < 0.01), and with the inclusion of control variables in Model 4, it is 0.067 (*p* < 0.01). These results indicate that both establishing health records and receiving health education have a significant positive impact on the settlement intentions of migrant workers.

Regarding control variables, migrant workers who have a wider range of movement tend to have lower settlement intentions in cities. Higher settlement intentions are associated with being married, having a higher education level, longer duration of mobility, higher average monthly household income, and larger household size. Age and gender do not significantly influence the settlement intentions of migrant workers.

### 4.2 Endogenous tests

#### 4.2.1 Instrumental variable estimation tests

To estimate using the IV-Probit model, suitable instrumental variables must be identified. These variables should be correlated with the improvement in public health service levels but should not directly influence the current urban settlement intentions of migrant workers. In this study, the diagnosis of chronic diseases (hypertension or diabetes) among migrant workers is used as an instrumental variable. This variable serves as a forward-looking indicator of basic public health service investment, significantly influencing subsequent efforts to enhance health record coverage and health education training. The worsening of chronic disease diagnoses among migrant workers prompts local governments to increase their public health service investments for the migrant population, generally without a direct correlation to the individual settlement intentions of these workers.

Table 3 presents the regression results based on the IV-Probit model. From the first-stage estimation results in columns (1) and (3) of the IV-Probit model, the diagnosis of chronic diseases among migrant workers has a significant positive impact on both public health education and health record management, indicating that the instrumental variable meets the relevance condition. The Wald test parameters for health record management and public health education in the IV-Probit model are 11.00 and 10.61, respectively, both significant at the 1% level. This suggests that public health education and health record management are endogenous variables, making the IV-Probit model’s estimates more reliable than those from the Probit model.

**Table 3:** Instrumental variable estimation tests.

Variables	(1)	(2)	(3)	(4)
	The first stage	The second stage	The first stage	The second stage
Follow-up and physical examination	0.084*** (0.012)		0.583*** (0.086)	
Health records		1.395*** (0.420)		
Health education				0.201*** (0.060)
Control	Yes	Yes	Yes	Yes
Observations	79,715	79,715	79,715	79,715
Wald (chi 2)	11.00***		10.61***	

Robust standard errors in parentheses. **p < 0.05, ***p < 0.01.

In the second-stage estimation results in columns (2) and (4) of the IV-Probit model, establishing health records and receiving health education both significantly enhance migrant workers’ urban settlement intentions. These findings are consistent with previous estimation results, confirming that the positive impact of public health services on the settlement intentions of migrant workers is robust, thereby further supporting Hypothesis 1.

#### 4.2.2 Propensity score matching

This study uses PSM to further examine the impact of establishing health records and receiving health education on the urban settlement intentions of migrant workers. Three matching methods were employed: nearest neighbor matching, radius matching, and kernel matching. Table 4 presents the average treatment effects (ATT) obtained from the PSM analysis. The ATT coefficients for establishing health records are 0.051, 0.052, and 0.057, respectively. For receiving health education, the ATT coefficients are 0.032, 0.041, and 0.029. All differences are significantly positive at the 1% level. These findings indicate that both establishing health records and receiving health education significantly enhance the urban settlement intentions of migrant workers, consistent with the basic regression results.

**Table 4:** PSM result.

Variables	Matching types	Treated	Controlled	ATT	S.E	T-value
Health records	K-nearest neighbor matching	0.529	0.478	0.051	0.004	11.69***
	Radius matching	0.529	0.471	0.052	0.004	14.95***
	Kernel matching	0.529	0.472	0.057	0.004	14.60***
Health education	K-nearest neighbor matching	0.496	0.464	0.032	0.004	7.11***
	Radius matching	0.495	0.455	0.041	0.004	9.99***
	Kernel matching	0.495	0.467	0.029	0.004	9.16***

Robust standard errors in parentheses. **p < 0.05, ***p < 0.01.

**Figure 1:**
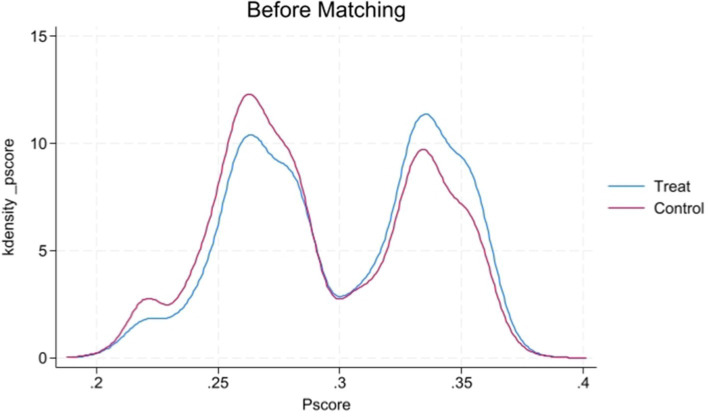
Kernel density plot of propensity scores before matching for health records.

**Figure 2:**
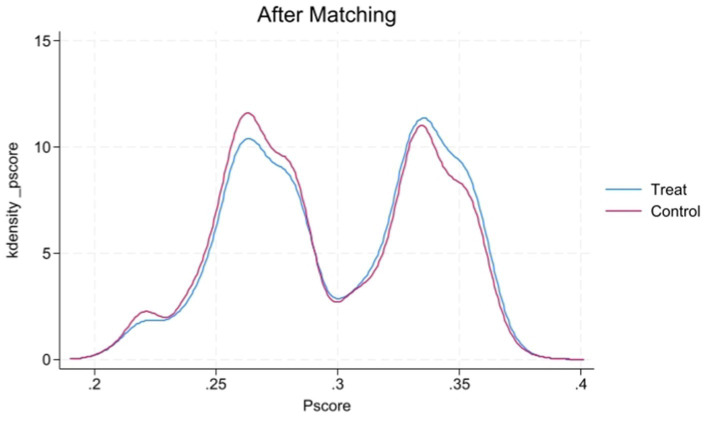
Kernel density plot of propensity scores after matching for health records.

**Figure 3:**
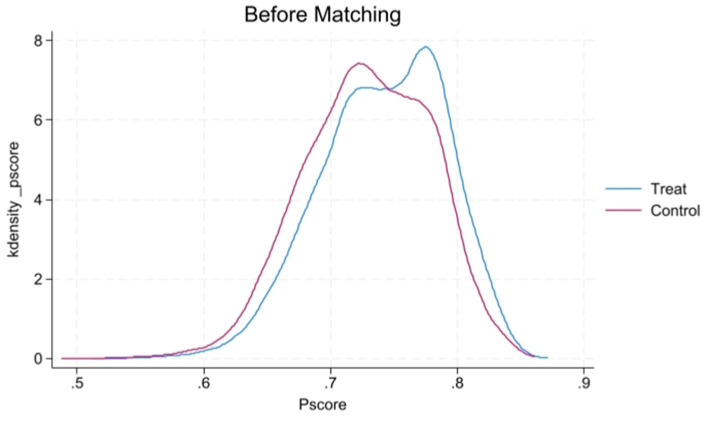
Kernel density plot of propensity scores before matching for health education.

**Figure 4:**
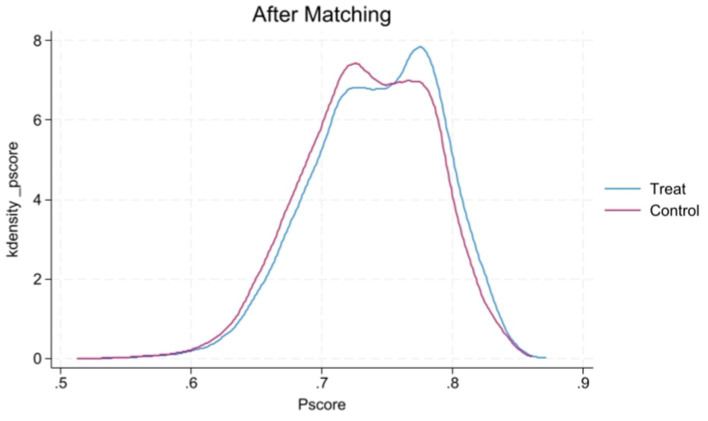
Kernel density plot of propensity scores after matching for health education.

Figures 1, 2 display the propensity score distributions for health records before and after matching, while Figures 3, 4 show the propensity score distributions for health education before and after matching. The matching method used in these analyses is one-to-one matching.

### 4.3 Robustness test

To further verify the robustness of the impact of establishing health records and receiving health education on the urban settlement intentions of migrant workers, we conducted robustness checks by substituting the dependent variable, using alternative analytical models, and adding control variables. The results of these robustness checks are presented in Table 5.

**Table 5:** Robustness checks.

Variables	(1)	(2)	(3)	(4)	(5)	(6)
	Dependent variable replacement		Logit model		Add control variables	
Health records	0.041*** (0.010)		0.197*** (0.016)		0.114*** (0.010)	
Health education		0.003*** (0.001)		0.110*** (0.017)		0.070** (0.011)
Control	YES	YES	YES	YES	YES	YES
Constant	−1.000*** (0.076)	−0.999*** (0.076)	−4.862*** (0.130)	−4.888*** (0.130)	−2.961*** (0.079)	−2.977*** (0.079)
Pseudo *R*^2^	0.012	0.011	0.069	0.068	0.074	0.073
Observations	79,713	79,713	79,713	79,713	79,713	79,713

Robust standard errors in parentheses. **p < 0.05, ***p < 0.01.

#### 4.3.1 Dependent variable replacement

To ensure the robustness of our findings, we replaced the urban settlement intention variable with the intention to transfer household registration as the dependent variable. The intention to transfer household registration reflects migrant workers’ willingness to change their residential registration, thereby indicating a long-term or permanent settlement in the destination city. Similar to the urban settlement intention variable, the intention to transfer household registration is set as a binary variable. The specific survey question was, “If you meet the local household registration requirements, are you willing to transfer your household registration here?” Responses of “yes” were coded as 1, and “no” were coded as 0. The estimation results are shown in columns (1) and (2) of Table 5, where columns (1) and (2) represent the impact of establishing health records and receiving public health education on migrant workers’ intention to transfer household registration, respectively. The results indicate that public health measures such as establishing health records and receiving public health education significantly enhance migrant workers’ intention to transfer household registration.

#### 4.3.2 Model replacement

To further verify the robustness of our experimental results, columns (3) and (4) of Table 5 report the effects of establishing health records and receiving health education on the settlement intentions of migrant workers using a binary Logit model instead of the Probit model. The impact coefficients are 0.197 (*p* < 0.01) and 0.110 (*p* < 0.01), respectively. These estimation results demonstrate that public health measures such as establishing health records and receiving public health education significantly enhance migrant workers’ intention to transfer household registration.

#### 4.3.3 Add control variables

Previous studies have found that land ownership is a significant factor influencing the settlement intentions of migrant workers (54). This implies that migrant workers who own contracted land in their hometowns are less likely to settle in the destination cities. Based on this, models 5 and 6 in Table 5 incorporate the control variable “land ownership status.” The specific survey question was, “Do you have a homestead in your registered hometown?” The regression coefficients for health records and health education are 0.010 (*p* < 0.01) and 0.070 (*p* < 0.01), respectively. The results indicate that the coefficients for health records and health education on the settlement intentions of migrant workers are significantly positive at the 1% level.

## 5 Further analysis

### 5.1 Explanatory mechanism

Given that the dependent variable in this study is binary, we use the KHB decomposition method (55) to examine the mechanisms through which public health services affect the urban settlement intentions of migrant workers. This method is suitable for situations where the dependent variable is discrete and has gained widespread application in recent research. We selected urban satisfaction and sense of belonging as mediating variables for our analysis. Urban satisfaction is measured by respondents’ agreement with the statement “I like the city/place where I currently live” from the CMDS questionnaire. Sense of belonging is assessed based on agreement with the statement “I am very willing to integrate with the local people and become one of them.” Responses are coded as 1 (Strongly Disagree), 2 (Disagree), 3 (Agree), and 4 (Strongly Agree). Table 6 presents the estimation results using the KHB method, illustrating how these mediating variables influence the relationship between public health services and the urban settlement intentions of migrant workers.

**Table 6:** Explanatory mechanism.

Variables	Health records		Health education	
	Urban satisfaction	Urban belonging	Urban satisfaction	Urban belonging
	(1)	(2)	(3)	(4)
Total effect	0.062*** (0.004)	0.061*** (0.004)	0.042*** (0.004)	0.041*** (0.004)
Direct effect	0.046*** (0.004)	0.040*** (0.004)	0.029*** (0.004)	0.023*** (0.004)
Indirect effect	0.016*** (0.001)	0.021*** (0.001)	0.013*** (0.004)	0.019*** (0.004)
Control	YES	YES	YES	YES
Observations	79,715	79,715	79,715	79,715

Robust standard errors in parentheses. **p < 0.05, ***p < 0.01.

From columns (1) and (3) of Table 6, we can see that the indirect effect of urban satisfaction is significantly positive at the 1% level. This indicates that establishing health records and providing public health education indirectly enhance migrant workers’ urban settlement intentions by improving their urban satisfaction. Similarly, columns (2) and (4) show that the indirect effect of sense of belonging is also significantly positive at the 1% level. This means that both establishing health records and receiving public health education increase migrant workers’ urban settlement intentions by enhancing their sense of belonging to the city. Further analysis reveals that the indirect effect of urban satisfaction accounts for 26 and 31% of the total effect of health records and public health education, respectively, on migrant workers’ urban settlement intentions. The indirect effect of sense of belonging constitutes 34 and 46% of the total effect of health records and public health education, respectively. These findings indicate that public health services can indirectly increase migrant workers’ urban settlement intentions by improving urban satisfaction and sense of belonging, with a greater indirect effect from sense of belonging. This further supports Hypothesis H2.

### 5.2 Heterogeneous analysis

Building on previous research and considering the impact of control variables on the dependent variable, this study conducts further heterogeneity analysis based on spatial and family dimensions. The analysis focuses on migrant workers with varying migration distances and marital statuses. Table 7 presents the results of the heterogeneity analysis.

**Table 7:** Heterogeneous analysis.

Variables	(1)	(2)	(3)	(4)
	The flow distance		Marital status	
	Cross provincial	Intra provincial	Married	Unmarried
Health records	0.154*** (0.015)	0.122*** (0.014)	0.139*** (0.011)	0.092*** (0.026)
Health education	0.116*** (0.014)	0.060*** (0.015)	0.103*** (0.011)	0.010** (0.003)
Control variables	YES	YES	YES	YES
Observations	40,281	39,432	66,549	13,164

Robust standard errors in parentheses. **p < 0.05, ***p < 0.01.

Columns (1) and (2) of Table 7 report the differences in the impact of health records and health education on the urban settlement intentions of migrant workers based on migration distance. The regression results indicate that the effects of establishing health records and receiving health education are more significant for migrant workers who move across provinces, with impact coefficients of 0.154 and 0.116, respectively, both significant at the 1% level. A possible explanation for this is that inter-provincial migration often involves greater challenges due to being far from home and family, as well as significant differences in lifestyle and culture. As a result, migrant workers who move across provinces face more difficulties in adapting to and integrating into urban society (56). In particular, in large metropolitan areas, this process represents an opportunity to shape urban life through access to various resources and opportunities. Establishing health records and receiving health education play a crucial role in helping migrant workers overcome these challenges, thereby significantly enhancing their settlement intentions in such cities (57). This situation requires greater attention and support from the city. Public health services such as health record management and public health education can precisely meet the needs of migrant workers, thereby enhancing their sense of belonging to the city and increasing their settlement intentions.

Columns (3) and (4) of Table 7 report the differences in the impact of health records and health education on the urban settlement intentions of migrant workers based on marital status. The regression results show that the effects of establishing health records and receiving health education are more significant for married migrant workers, with impact coefficients of 0.139 and 0.103, respectively, both significant at the 1% level. From a family perspective, the settlement decisions of migrant workers are closely related to each family member. When choosing to migrate to a city, migrant workers aim to maximize expected benefits while minimizing risks to the family. Public health measures like health record management and health education can provide the necessary support and stability, making it easier for married migrant workers to settle in urban areas (58). For married migrant workers, the establishment of a marital relationship allows both partners to share life risks, which helps alleviate the pressures of living in the destination city (59). In this context, the impact of public health services on one partner’s settlement intentions is likely to be transmitted through social relationships to the other partner. This finding aligns with previous research, which suggests that migrant workers who move together with family members (spouse, children) or in pairs tend to stay longer in the destination city compared to those who move alone (60). Moreover, the migration structure of migrant workers is gradually shifting from individual migration to family migration.

## 6 Conclusions and implications

Based on CMDS2017 data, this study empirically examines the impact of public health services, such as establishing health records and public health education, on the urban settlement intentions of migrant workers using a probit model. Furthermore, the study explores the mechanisms and heterogeneity of these effects. As one of the developing countries with the largest migrant population in the world, the findings of this research can help optimize the implementation of public health service policies and provide meaningful guidance for enhancing the urban integration of migrant workers.

The results of this study indicate the following:

First, as key aspects of public health service provision, establishing health records and receiving health education have substantially increased migrant workers’ urban settlement intentions. The impact of establishing health records is even more pronounced. This conclusion remains robust after being tested through instrumental variable methods, propensity score matching, substituting dependent variables, modifying analytical models, and adding control variables.

Second, public health services have a greater positive impact on the settlement intentions of migrant workers who move across provinces and those who are married.

Third, the mechanism analysis reveals that public health services can indirectly influence migrant workers’ urban settlement intentions through urban satisfaction and urban belonging, with the latter having a stronger indirect effect.

Based on these findings, the following policy implications can be considered to enhance urbanization processes and improve the efficiency of human capital allocation.

First, expand the coverage of electronic health records. Enhance the management of health records and promote related public awareness and guidance to increase the knowledge and utilization of public health services. In the context of the current digital transformation of public health services, efforts should be made to improve the creation rate of electronic health records and consequently improve health levels. Standardize data across health records and eliminate discrepancies in data standards among medical institutions and other government departments. This will lay a solid foundation for the widespread use of electronic health records, thereby further increasing the urban settlement intentions of migrant workers and other internal migrants.

Second, improve the quality and specificity of health education. Move away from traditional, uniform, and standardized education content and methods by tailoring public health education to local conditions. Considering the migration characteristics of migrant workers, focus health education efforts on the communities where they reside. Enhance the specificity of educational content, with particular emphasis on public health education activities targeting married migrant workers and those who have migrated across provinces.

Third, enhance social integration levels for migrant workers. Social integration is a key factor influencing the urban settlement intentions of migrant workers, necessitating further enhancement of public health services to boost urban satisfaction and urban belonging. Given that communities are the fundamental units of urban life, they should be the primary arenas for social interaction. Develop and strengthen community services and management systems that cover migrant workers, ensuring that communities effectively facilitate social integration. Foster a friendly and supportive community atmosphere to help migrant workers better integrate into the city, thereby increasing their sense of belonging and identification with the urban environment.

## Data Availability

The original contributions presented in the study are included in the article/supplementary material, further inquiries can be directed to the corresponding author.
